# Can the Part Replace the Whole? A Choice Experiment on Organic and Pesticide-Free Labels

**DOI:** 10.3390/foods11172564

**Published:** 2022-08-24

**Authors:** Qiuqin Zheng, Xiaoting Wen, Xintian Xiu, Xiaoke Yang, Qiuhua Chen

**Affiliations:** 1 College of Economics and Management, Fujian Agriculture and Forestry University, Fuzhou 350002, China; 2 College of Resource and Environment, Fujian Agriculture and Forestry University, Fuzhou 350002, China; 3 School of Humanities, Fujian University of Technology, Fuzhou 350002, China

**Keywords:** organic labels, pesticide-free, choice experiment method, willingness to pay, green tea, consumer preference, eco-label

## Abstract

Chemical pesticides are a serious impediment to agricultural sustainability. A large-scale reduction in their use to secure food supplies requires more innovative and flexible production systems. Pesticide-free production standards bring together the strengths of all participants in the food value chain and could be the catalyst for this transition. Using a choice experiment approach and green tea as an example, this study investigated consumers’ preferences for organic and pesticide-free labels. According to the findings, organic and pesticide-free labels and brands are all major factors that affect consumers’ purchase decisions. Consumers are more willing to pay for organic labels than pesticide-free labels. There is a substitution effect between organic labels and pesticide-free labels. Complementary effects exist between organic labels and national brands, pesticide-free labels, and national brands. Consumer trust has an impact on consumers’ choice of organic labels and pesticide-free labels. The use of pesticide-free labels is an alternate approach for small- and medium-sized businesses in a specific market to lower the cost of organic certification.

## 1. Introduction

A critical attribute of food safety is pesticide residue [1]. Using chemical pesticides can significantly increase food production and improve agricultural efficiency, but it also causes damage to the natural ecological environment and the quality and safety of agricultural products [2]. The overuse of chemical pesticides can lead to the rapid growth of resistance in target pests, as well as serious impacts on non-target organisms, for example, endocrine disorders in rats, birds, and fish [3]. Pesticide residues can spread throughout the environment, contaminating different ecosystems and damaging food and water resources. Examples include high nitrate levels in groundwater, reduced soil fertility, and increased greenhouse gas emissions [4]. Chemical pesticides are considered to be one of the most prominent barriers to agricultural sustainability [3]. Pesticide risk reduction is at the top of many countries’ policy agendas, but most have failed to meet their targets [5]. Existing policies often fail to promote widespread adoption of pesticide-free production practices due to the lack of cost-effective alternatives [6]. The vigorous development of organic agriculture is one approach to addressing the problem of agricultural products quality and safety [7]. Organic certification requires attributes such as no chemically synthesized fertilizers, pesticides, growth regulators, or other substances, and no pesticide residue, growth hormones, or genetic engineering [8].

In China, the organic food market is rapidly expanding and has reached a considerable size. Nonetheless, the share of available organic food remains small [9]. According to the Global Organic Agriculture Statistical Yearbook 2020, global sales of organic food and drinks exceeded EUR 95 billion in 2018. Of this, China’s organic food sales were EUR 8.1 billion, accounting for only 8.3% [10]. Organic farming production in China necessitates a 3-year conversion period and increased labor expenditure [11]. Despite the potential premium, organic agricultural products incur higher production costs than conventional agricultural products and require significant investment, which many Chinese small- and medium-sized businesses (SMEs) cannot afford [12]. For consumers, the high cost of organic production leads to higher prices for organic agricultural products, which has hindered many consumers from buying [13].

Large-scale reductions in pesticide use in the context of unfavorable food production require more innovative and flexible systems to complement organic farming [14]. Pesticide-free production standards, which combine the strengths of all food value chain players, may be the cornerstone of this shift [15]. In Switzerland, the IP-SUISSE producer organization is introducing a nonorganic, private–public standard for pesticide-free wheat production [15]. Studies have demonstrated that the pesticide-free attribute is the most important aspect of consumer interest when purchasing organic produce [16,17]. The study by Britwum, et al. [18] on consumers’ perceptions regarding the desired attributes of organic produce found that consumers place the highest importance and confidence in the “free of growth hormones” and “free of synthetic pesticides” aspects of organic labeling. For Chinese consumers, purchasing organic agricultural products is motivated more by concerns regarding food safety and personal health and less by environmental protection [19,20]. Generally, institutional pesticide-free certification is less difficult and less costly to achieve than certified organic labeling. Do consumers prefer separate pesticide-free information? If consumers are willing to pay for separate pesticide-free information, SMEs can use such certification without assuming the prohibitive expenditure of converting to organic operations. For SMEs, pesticide-free information could offer a strategic alternative to give farmers a competitive advantage. Consumers will then be able to buy healthy and safe products at a lower cost. Hence, investigating consumers’ preferences and willingness to pay (WTP) for organic labels and pesticide-free information will directly affect agricultural certification decision-making.

A series of studies have been conducted on consumers’ preference, WTP, and the influencing factors of organic labels [13,21,22,23,24]. Regarding how consumers perceive pesticide-free attributes, scholars believe that previous research has not been systematic and in-depth enough [18,25]. Bernard and Bernard [26] examined the WTP for two core attributes of organic labeling (pesticide-free and non-GMO), finding that consumers were willing to pay for the pesticide-free information. By contrast, Edenbrandt [25] surveyed Danish consumers and found that pesticide-free information was less important to consumers than the organic label, indicating that Danish consumers preferred to buy organic produce. These contradictory findings warrant further investigation.

Tea is one of the three most recognized drinks worldwide. China is the largest tea-producing country and a major tea-consuming and exporting country in the world [27]. Green tea production accounted for 61.70% of the total tea production in 2020. The export volume of green tea is 293,400 tons, accounting for 84.1% of China’s total tea exports [28]. With consumers’ increasing concerns regarding the quality of life and the rising threshold of international trade in tea, the production of organic green tea represents an important approach for enhancing the competitiveness of green tea in China, promoting green tea export, and expanding domestic demand for green tea. Existing literature focuses on the organic consumption behavior of milk [29,30,31], rice [32], and other crops [33,34], but there are fewer studies on the organic consumption behavior of tea [35]. Thus, green tea was chosen as the experimental subject in this study.

The choice experiment (CE) method can estimate consumers’ preferences for different product attributes and assess the relationships between attributes. It avoids the limitations of the contingent valuation method that can only measure a single attribute of a product [35]. Based on the above background, taking green tea as an example, this study applies the CE method to analyze the following questions: (1) Under current conditions, do Chinese consumers have a preference and WTP for organic and pesticide-free labels? (2) Are pesticide-free labels valid in comparison with organic labels? (3) What are the factors that influence consumers’ WTP for organic and pesticide-free labels? This study can provide valuable information for market expansion and marketing of organic agricultural products and also reduces the degree of information asymmetry between SMEs and consumers, providing a reference for SME producers to control production costs.

## 2. Materials and Methods

### 2.1. Attribute Selection

The CE method is widely used to measure product preferences and is an excellent approach for estimating multiple attributes. Attribute selection is the basis for determining the validity and precision of the results [36]. Previous studies have shown that food safety attributes and brands are crucial to consumers’ preferences in green tea [34]. This study assumes that green tea is a collection of organic labels, pesticide-free labels, brands, and prices. Table 1 presents the attributes and levels.

Organic labels are widely evident in the real market. Tea companies use organic logos in product packaging to distinguish products from conventionally produced teas. There are currently no certified pesticide-free labels in tea packaging, and only some e-commerce tea companies present reports confirming pesticide-free status on product details pages. To highlight the pesticide-free characteristic and facilitate respondents’ understanding, this study used a simplified logo to represent pesticide-free status, referring to Grebitus, et al. [37]. The pesticide-free label used in this study refers to the green tea are grown without chemical pesticides, herbicides, or synthetic fertilizers.

The brand is also an important factor in consumer decision-making. The brand is a “search attribute” that serves as an extrinsic factor to signal and enhance consumers’ trust [38]; thus, consumers are willing to pay a higher price premium for preferred brands [34,39]. The cultivation and promotion of brand identity can motivate green tea producers to improve and optimize product quality. From the perspective of SME tea producers, branding should be vigorously established and promoted. Generally speaking, national brands are considered to have higher quality and safety than regional brands [35,40]; however, different tea drinking habits exist in different regions, and the effect of teas’ origins is extremely prominent [41,42]. Hence, regional brands may be more easily accepted by local consumers [35]. Previous studies have conducted investigations regarding geographical indications or origins [42], but few studies have analyzed both national and regional brands.

Price is one of the most significant factors in consumers’ purchase decisions. To set realistic price levels, this study averaged the prices of the top 50 bulk green teas sold on Taobao. Given the considerable premium for green tea in gift boxes, only green tea in bags is used. It should be noted that the green tea set up in this study does not exactly exist in the real market. Generic green tea can be considered as the lowest level of hypothetical green tea varieties [43]. Therefore, the final average price of green tea was set at 101 RMB/500 g, and the other three levels are set at 10%, 15%, and 20% higher.

### 2.2. Experimental Design

According to the settings in Table 1, a total of 2 × 2 × 3 × 4 = 48 dummy scenarios are generated in the full-factorial experimental design. If each choice set contains two different green tea profiles, respondents will face 2256 choices. Considering the cost and feasibility, this study applied a D-optimal design, as it can ensure validity (D-efficiency) while reducing the asymptotic standard error among attributes [44]. After D-optimal using Negene 1.0 software, a final set of 36 options was randomly generated with a D-efficiency of 93.73%, a D-error of 0.089, and an A-error of 0.103.

**Figure 1:**
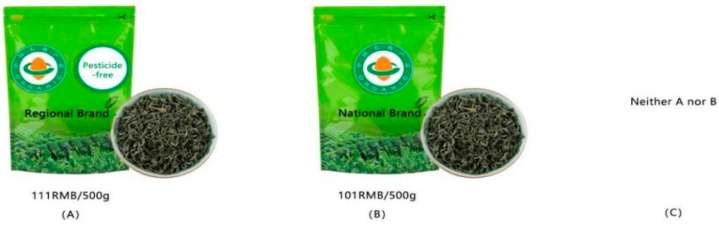
Example of a choice set. Note: (**A**–**C**) in the figure means the alternative in the choice set.

According to Kessels et al. [45], due to consumers’ limited information load capacity, the number of consumer choices is appropriate at eight. Thus, 36 choice sets were assigned to six versions of the questionnaire, and each version of the questionnaire contained six choice sets. Following Wu, et al. [46], “neither option A nor option B” was included to simulate purchase circumstances more realistically. Hence, each choice set contained two virtual green tea product sets and one “neither option A nor option B.” Figure 1 shows an example of the choice set.

Several studies have argued that trust would affect consumers’ preferences [47]. Low trust is associated with lower ratings of the label itself, which further reduces purchase intention [48]. Two kinds of labels were set in this study. Referencing Wu [49], this study established items of consumers’ trust in organic labels and pesticide-free labels. These items were scored using a five-point Likert scale from 1 for “absolutely disagree” to 5 for “absolutely agree.” Table 2 presents the detailed items.

### 2.3. Sample Size Determination

The rule of thumb is usually used to calculate the required sample size. The minimum sample size is determined by a combination of three factors: the number of choice sets (*t*), the number of alternatives (*a*), and the maximum number of levels (*c*)of the attribute [50,51].

(1) $$N>\frac{500\times c}{t\times a}$$

Hence, the minimum sample size of this study is 500 × 4 ÷ 6 ÷ 3 = 111. Furthermore, according to Yamane (1967) [52,53], the minimum sample size in the study should be:

(2) $$n=\frac{Z^{2}p\left(1-p\right)}{e^{2}}=\frac{1.96^{2}\times 0.5\times \left(1-0.5\right)}{0.05^{2}}=384.16$$

where *Z* is the significance level of 95%, the value of the distribution table *Z* = 1.96, *p* is the estimate of the correct prediction of *n* for *p* = 0.5, *e* is the sampling error allowed with +/−0.05 (5%). It is noted that the sample size calculated according to the formula is the minimum sample size suggested due to the requirement for stability of the utility estimates. In the actual research situation, the required sample size is larger than the minimum value.

### 2.4. Data Collection

For respondent selection, actual consumers of the product should be selected as the target, as only respondents who are familiar with green tea will be concerned about the various attributes [54]. According to Determann, et al. [55], no significant difference was found between online and offline surveys for consumers’ preferences in CE; hence, this study used an online survey.

We chose the Questionnaire Star platform (a professional online survey company) to conduct the online survey. The Questionnaire Star sample base is widely sourced and covers a wide range of consumer groups of different ages, occupations and income levels. It is widely used in consumer preference research [56]. As a commissioned network survey, the respondents are generally randomly selected by the commissioned company in its sample database through the network system. To ensure that the respondents identified by random selection met the requirements of this study, the following controls were also conducted in this study. (1) By setting the sample filter question before the formal questionnaire responses: “Have you purchased green tea in the last year?” (2) Screening of targets by age information in the sample pool. This ensures that the participants in the choice experiment survey are real consumers who are at least 18 years old and have had experience in purchasing green tea. Additionally, this study set a validation question [57], “Please select the ‘red’ option from the following options.” Respondents who chose another color were direct to the end of the surveys. A total of 430 valid questionnaires were returned, and Stata 16.0 was used to calculate the final questionnaire data.

The questionnaire consisted of three parts: (a) consumers’ trust; (b) comparing alternatives in CE; (c) respondents’ socio-demographic characteristics. Given that CE is a hypothetical experiment, hypothetical bias may be present. Referencing Tonsor and Shupp [49], this study presented a brief introduction to respondents, using pictorial examples and textual descriptions of organic labels and pesticide-free labels. After this, two multiple-choice questions were set in this study: “Which of the following characteristics does the organic label contain?”, “Which of the following characteristics does the pesticide residue-free label contain?” Only those who choose both correct questions are considered valid. This ensures that respondents understand the meaning of organic and pesticide-free labels before conducting the CE.

### 2.5. Models

Based on the consumer utility theory proposed by Lancaster (1966), the utility perceived by consumers from a product does not come from the product itself but from its attributes; thus, in the discrete choice model, the utility obtained by consumer *n* for choice *i* is expressed as follows:

(3) $$U_{nit}=V_{nit}\left(\beta_{n}\right)+\varepsilon_{nit}=\delta \left(ASC\right)+\alpha_{n}\left(X_{i}\right)+\gamma_{n}\left(-P_{i}\right)+\varepsilon_{nit}$$

where *U_nit_* is the utility obtained by consumer *n* from choice *i* in choice set *t*, *V_nit_* (*β_n_*) is the observable utility of parameter *β_n_*, and *ε_ni_* represents a random error. *V_nit_ (β_n_)* consists of three parts. ASC is the specific choice constant. When ASC is 1, it indicates that the respondent chooses the “opt-out” option. *X_i_* is the factor that affects the observable utility *V_nit_*, which includes the product attributes and the respondent’s characteristics *n*. *P_i_* is the retention utility, which represents the premium paid for a change in *X_i._ β_n_* = (*δ, α_n_, γ_n_*) is a vector of parameters reflecting respondents’ ASC preferences and other attributes.

In this study, the main effect of the attributes was determined using Equation (4). Organic label (ORG), pesticide-free label (PEST), regional brand (RGB), and national brand (NAB) were the categorical variables, and the “none” label was used as the baseline. Price was the metric variable in accordance with the four price levels designated in the experiment. The utility function model is expressed by Equation (4):

(4) $$U_{nit}=ASC+\beta_{1}Price_{nit}+\beta_{2n}ORG_{nit}+\beta_{3n}PEST_{nit}+\beta_{4n}RGB_{nit}+\beta_{5n}NAB_{nit}\;+\varepsilon_{nit}$$

where ASC is the “opt-out” option and the coefficients from *β*_1_ to *β*_5*n*_ are the parameter vectors of the attributes estimated.

For the interaction effects of the attributes, organic trust (OTRU) and pesticide-free trust (PTRU) were the explanatory variables representing consumer trust in organic labels and pesticide-free labels, respectively. Indices of these two attitudinal variables were created by the mean values of the item scores. The utility function with interaction is expressed by Equation (5):

(5) $$U_{nit}=ASC+\beta_{1}Price_{nit}+\beta_{2n}ORG_{nit}+\beta_{3n}PEST_{nit}+\beta_{4n}RGB_{nit}+\beta_{5n}NAB_{nit}+\varepsilon_{nit}\;+\beta_{6n}\left(ORG_{nit}\times OTRU_{n}\right)+\beta_{7n}\left(PEST_{nit}\times OTRU_{n}\right)+\beta_{8n}\left(RGB_{nit}\times OTRU_{n}\right)\;+\beta_{9n}\left(NAB_{nit}\times OTRU_{n}\right)+\beta_{10n}\left(ORG_{nit}\times PTRU_{n}\right)+\beta_{11n}\left(PEST_{nit}\times PTRU_{n}\right)\;+\beta_{12n}\left(RGB_{nit}\times PTRU_{n}\right)+\beta_{13n}\left(NAB_{nit}\times PTRU_{n}\right)+\varepsilon_{nit}$$

Consumer *n*’s WTP for attribute *x* is estimated by Equation (6):

(6) $$WTP_{n}=\beta_{nx}/\beta_{np}$$

## 3. Results

### 3.1. Socio-Demographics of Consumers

Table 3 presents the socio-demographics of the respondents. Among the final sample of 430, there was a slightly higher number of female respondents (54.46%) than male ones (45.54%). This is consistent with some previous studies wherein females are the primary household buyers [58]. Respondents aged 25–34 years hold the largest share (59.90%), followed by those aged 35–44 years (16.34%). Although middle-aged consumers are the main buyers of green tea, the rise of younger consumers cannot be ignored. The married samples were predominant, and most of them had some college or a bachelor’s degree. Respondents with a monthly household income of 14,000 RMB and above occupied the largest proportion (30.94%), followed by those with 10,000–11,999 RMB and 12,000–13,999 RMB monthly household income. The higher monthly income and education may be because the study targeted consumers who had purchased green tea. According to Chen, et al. [59], tea consumption is positively correlated with consumers’ income. Almost all of the respondents had more than three people living together. Additionally, 70.3% and 54.21% of the respondents had children aged 12 and below and elderly aged 65 and above, respectively. In terms of tea consumption frequency, the percentage of respondents who purchased green tea once every 1–2 months was 68.56%.

### 3.2. Main Effect

Using the mixed logit model, this study set price and its cross terms as fixed parameters, and other attribute variables are set as random parameters. The log-likelihood values of the mixed logit model (−1629.2003 and −1619.7091) indicate that the regression results are generally significant.

Table 4 presents the results of the mixed logit model. In the main effects model, the parameters of the selected attributes are regressed to elicit the consumer preferences for attributes of the organic label, pesticide-free label, regional brand, and national brand. The results of the model estimation show a log-likelihood of −1629.2003, and the regression results are generally significant. The specific alternative constant ASC is significantly negative at the 1% level, indicating that choosing “neither A nor B” has a negative effect on consumer utility when compared with the combination of green tea attributes offered in the study. All of the green tea attribute combination options offered in this study could increase consumer utility. Price is negative and significant at the 5% level, indicating that consumers prefer lower-priced products. The higher the price of green tea, the more negatively it affects consumer utility. The three organic, pesticide-free, and national brand labels are significantly positive at the 1% level, indicating that consumers hold a positive preference for these three labels. The parameter estimation of different labels reveals that consumers have the highest preference for the organic label (1.282), followed by pesticide-free label (0.662) and national brand (0.459).

In the main effect with the interaction model, the variable “ORG × PEST” is significantly negative at the 10% level, indicating that there is a substitution effect between the organic label and pesticide-free label. The variables “ORG × NAB” and “PEST × NAB” are significantly positive at the 10% level and the 1% level, respectively. When the organic label or the pesticide-free label is attached to the national brand, consumers’ utility is enhanced.

### 3.3. Main Effect with Interaction in Trust

This section investigates the conjoint effect of trust in the organic and pesticide-free with the given attributes. Two averaged indices in Table 2 were used in a conjoint regression. The results are shown in Table 5.

The interaction term between organic trust and the organic label and regional brand is significantly positive. This indicates that the more consumers show trust in organic labels, the more they prefer organic labels and regional brands.

The interaction term between pesticide-free trust and the organic label, the pesticide-free label is significantly positive. This indicates that those who trust in pesticide-free will prefer organic labels too. Pesticide-free is an important attribute of organic labels. The interaction term between pesticide-free trust and the regional brand is significantly negative.

### 3.4. Heterogeneity Analysis Considering Other Consumer Factors

Heterogeneity exists in consumer preferences for organic and pesticide-free labels. To analyze the sources of heterogeneity, interaction terms of socio-demographics and consumption habits with each attribute of green tea were introduced in the model. Table 6 presents the results.

Considering socio-demographics, sex, household size, and income have a significant impact on the preference for organic labels. The “education × PEST” variable is significantly positive, while the “elder × PEST” variable is significantly negative. This implies that green tea with a pesticide-free label could attenuate the utility of consumers with elderly people over 65 years of age at home. The “income × RGB” and “children × RGB” are significantly positive, indicating that higher income consumers and those who with children under 12 years of age at home are more likely to buy green tea with a regional brand. Conversely, female consumers are more likely to buy green tea from a national brand. In addition, females, older, bigger household sizes, and consumers with children under 12 years of age at home are rather to choose the opt-out option. They might tend to keep the status quo.

### 3.5. Willingness to Pay

WTP can directly reflect the change in consumer utility when each attribute changes. The Hierarchical Bayes (HB) approach [60,61] was introduced in this study. Estimations were computed in Stata 16.0 using the command Bayesmixedlogitwtp developed by Baker [62]. Some studies have already used HB to estimate discrete choice models [63,64]. Table 7 shows the results.

In terms of magnitudes, Chinese consumers have highly valued the organic label, with a mean WTP of 148.9 RMB/500 g among all attributes. Chinese consumers also showed a positive preference for the pesticide-free label with a mean WTP of 87.1 RMB/500 g. The reason may be that compared to pesticide-free labels, organic labels include not only food safety attributes (e.g., “no pesticide residue”) but also environmental value attributes (e.g., “good for biodiversity” and “low pollution”) [65]. In addition, the mean WTP for a national brand is 40.6 RMB/500 g.

Relative to the market price (101 RMB/500 g), the premium for the organic label reached 47.43%. In real life, the price premium of organic green tea over conventional green tea is approximately 50%, indicating that the WTP for organic green tea must be further improved.

## 4. Discussion

Chinese consumers’ demand and preference for safer food have increased significantly because of health concerns [19]. This study confirms that both organic and pesticide-free labels can increase Chinese consumers’ perceived utility. This finding is consistent with other studies [66,67], i.e., Chinese consumers have a positive preference for organic food. Organic labels contain not only health and safety attributes but also eco-attributes, such as being environmentally friendly. As society evolves and consumer environmental awareness rises, a growing number of Chinese consumers are motivated by environmental beliefs when buying organic products [68]. Researchers have compared consumer preferences for organic and pesticide-free labels in previous studies. Bernard and Bernard [26] examined consumers’ preferences and WTP for organic, pesticide-free, non-GMO, and general products. They found no significant difference in consumer preferences between the organic label as a whole and its parts, and a strong substitution relationship between the whole and its parts. Consumers’ WTP for the organic label as a whole is found to be greater than the WTP for each part individually. Grebitus, Peschel, and Hughner [37] examined U.S. consumers’ preferences and WTP for pesticide-free labels using Medjool dates, finding that U.S. consumers had positive preferences for pesticide-free labels and were willing to pay more. By contrast, Edenbrandt [25] used rye bread as a subject, asserting that the pesticide-free label was not valuable and that people would only buy organic bread. This study demonstrates that the pesticide-free label is considered valuable on its own by Chinese consumers. The possible reason for this result is health concerns. Roos and Tjarnemo [69] noted that consumers were more concerned with attributes related to personal interests than other long-term benefits. Thogersen, et al. [70] confirmed that the positive attitude of Chinese consumers toward organic food is primarily motivated by consumers’ concerns regarding the health value of organic food. Farias [71] demonstrated that the level of information on pesticide-free labels affected consumer preferences. As Chinese consumers become increasingly concerned about food quality and safety and health benefits, the pesticide-free label presents pesticide-related information more directly and visibly than the organic label, so that consumers have a clearer understanding of the quality and value of pesticide-free products. To sum up, both organic and pesticide-free labels have heterogeneous consumer groups and should be targeted to build markets according to their different attributes.

In real life, merchants will attach labels or additional features to goods to enhance the utility of the product itself and further gain more profits [72]. However, there is no unanimous conclusion in the academic community as to whether multiple labels necessarily enhance the utility of a product. Wang, et al. [73] proposed that consumers have a higher willingness to pay for food with both organic food and drug-free labels than organic food alone. The reason is that the more labels a food has, the more likely consumers believe the food is safer. The same idea also appears in Gabaix and Laibson [66,74] and Bertini, et al. [75] who propose that based on the quantity effect, consumers always perceive products with more attributes as superior to fewer attributes. However, Meas, et al. [76] proposed that whether more or fewer labels are better is not in the quantity but in the interaction between labels. He classified the interactions of labels into complementary effects and substitution effects. Several previous studies have shown a strong substitution effect between organic and pesticide-free labels [26]. The finding of this study is consistent with them. The organic label also contains the attribute of no pesticide residues, and there is a partial overlap in reflecting the value of the product; therefore, the overall value estimate for both labels will be less than the sum of the value estimates for the individual labels. Therefore, both labels need to be examined carefully and labeling decisions should not be based solely on the cost-benefit profile of a single label. In addition, this study also found a significant positive interaction effect between national brands and both organic and pesticide-free labels, showing strong complementary effects. According to Parguel et al. [77], brands can also act as a quality signal, and a high level of brand equity can represent a high level of product quality. National brands have higher visibility and better brand images than regional brands, and they can reflect the food quality from another perspective. When they are put together with the organic labels or pesticide-free labels, it does produce a one-plus-one effect. Compared with weak brands, strong brands are more likely to benefit from organic or pesticide-free labels. Therefore, well-known Chinese tea companies are encouraged to participate in organic label certification and to develop organic agriculture.

Consumers’ trust in labeling is also a new issue in the area of study [78,79]. The interaction terms demonstrated consumer trust has a positive effect on enhancing label preferences. This finding is consistent with those of studies [32,80]. In an earlier study, Yin, et al. [81] revealed a large level of consumer distrust in organic labels; however, in recent years, with the continuous promotion of the Chinese government and the market, consumer perceptions of organic labels have increased significantly. There is also a deeper understanding and awareness of the connotations of organic labels, which also drives consumer preference for pesticide-free labels. This study also examined the role of socio-demographics in choice. Age, marriage, and green tea purchase frequency had almost no effect on the purchase of green tea. Consumers who were female, had high income, had a large household size, and had elderly above 65 years old at home were more likely to purchase organic green tea. Those with higher education were more willing to purchase pesticide-free green tea. Females, older, larger household sizes, and consumers with children under 12 years old in the household were more likely to maintain the status quo. However, socio-demographics alone are not sufficient to explain the differences in consumer behavior and more intrinsic factors such as consumer psychology should be considered [82].

This study has some research limitations. First, the CE method used provides consumers with a given product profile, and consumers who are not price sensitive may bias the results, which can be further demonstrated in the future by incorporating methods such as random Nth-price auction experiments. Second, China is the largest tea-producing country, with significant tea export and trade. To meet the expectations of different countries, tea producers will often put organic labels of other countries on their packaging, such as the EU, Japan, or Brazil; hence, the type of label preferred by consumers is also a potential consideration for future study.

## 5. Conclusions

This study focused on consumer preferences for organic labels and pesticide-free labels among Chinese consumers. The research chose green tea, a real product in the organic market to conduct the CE. It was confirmed that Chinese consumers have preferences for organic labels, pesticide-free labels, regional brands, and national brands. The highest premium for selected attributes was about 39.83% for organic labels, followed by pesticide-free labels (20.58%), and national brands (14.26%). In addition, this study also confirmed a substitution effect between the organic labels and pesticide-free labels; a complementary effect between organic labels and national brands, pesticide-free labels, and national brands. Trust was considered and found that consumers with higher scores in trust preferred green tea with organic labels or the regional brand. The socio-demographics were used to analyze the heterogeneity in consumer preferences. Female and consumers with higher income prefer organic green tea, and consumers with higher education prefer green tea without pesticide residues. Household size and whether there are elderly above 65 or children under 12 in the family also affect the preference. Conversely, age, marriage, and green tea purchase frequency have almost no effect on green tea purchase.

The findings of this paper yield several practical insights. First, considering that the pesticide-free label is not currently in use, such labeling may offer a viable alternative to effectively reduce the costs paid by SMEs for organic certification. For marketers, knowing consumers’ preferences for pesticide-free attributes can also improve marketing strategy. For example, in certain markets, product packaging may consider using a pesticide-free label. Second, consumers have shown a highly positive preference for organic green tea, especially for when the organic label is placed alongside a national brand. Tea producers of well-known brands are encouraged to shift to sustainable production and organic certification to generate profits. Finally, trust is something that can contribute to the growth of organic green tea consumption. The government should adopt a responsible attitude and strengthen monitoring efforts to reduce food scandals, thus increasing consumer trust in organic food.

## Figures and Tables

**Table 1:** Green tea attributes and respective levels.

Attributes	Levels
Organic label	None; Organic label
Pesticide-free label	None; Pesticide-free label
Brand	None; Regional brand; National brand
Price	101 RMB/500 g; 111 RMB/500 g; 116 RMB/500 g; 121 RMB/500 g

**Table 2:** Characteristics of consumer trust in organic label and pesticide-free label.

Variable	Items	Mean	SD
Organic Trust	I trust in the certification process of organic labels	4.153	0.646
	I trust that the organic green tea on the market is produced according to organic standards	3.965	0.844
	If I see the organic label on the front of the package, I will trust that the product is organic	4.027	0.781
Pesticide-free Trust	I trust in the certification process of pesticide-free labels	3.903	0.828
	I trust that the pesticide-free food on the market is produced according to pesticide-free standards	3.854	0.871
	If I see the pesticide-free label on the front of the package, I will trust that the product is pesticide-free	3.767	0.899

Notes: SD = standard deviation.

**Table 3:** Sociodemographic characteristics of the sample (*n* = 430).

Variable	Definition	Percentage (*n* = 430)
Gender	Male	45.54%
	Female	54.46%
Age	18–24	19.80%
	25–34	59.90%
	35–44	16.34%
	45–54	3.22%
	55–64	0.50%
	≥65	0.25%
Marriage	Married	72.03%
	Unmarried	27.97%
Education	Junior high school or below	0.25%
	High school	3.96%
	College or bachelor’s degree	83.91%
	Post-graduate degree	11.88%
Members of household	≤2	8.17%
	3	40.84%
	4	28.47%
	≥5	22.52%
Are there children aged 12 and under in the household?	Yes	70.30%
	No	29.70%
Are there elderly people aged 65 and above in the household?	Yes	54.21%
	No	45.79%
Monthly household income (RMB)	≤2000	2.97%
	2001–3999	4.21%
	4000–5999	8.17%
	6000–7999	7.92%
	8000–9999	13.12%
	10,000–11,999	16.58%
	12,000–13,999	16.09%
	≥14,000	30.94%
Frequency	Once every half a month	13.86%
	Once a month	40.84%
	Once every 2 months	27.72%
	Two months and more	17.57%

**Table 4:** Results of the mixed logit model.

Attributes	Main Effect		Main Effect with Interaction	
	Coefficient	SD	Coefficient	SD
PRICE	−0.0319 ***	0.005	−0.0258 ***	0.005
ORG	1.282 ***	0.055	1.576 ***	0.254
PEST	0.662 ***	0.054	0.923 ***	0.255
RGB	0.248 ***	0.073	0.332	0.238
NAB	0.459 ***	0.072	−0.0914	0.210
ASC	−4.385 ***	0.560	−3.602 ***	0.589
ORG × PEST	−	−	−0.710 *	0.380
ORG × RGB	−	−	0.0116	0.254
ORG × NAB	−	−	0.432 *	0.252
PEST × RGB	−	−	-0.204	0.250
PEST × NAB	−	−	0.696 ***	0.249
χ^2^	1238.12		1226.94	
P	0.0000		0.000	
Log-likelihood	−1629.2003		−1619.7091	

Notes: * and *** indicate significance at the 10% and 1% levels, respectively. ASC = opt-out option; ORG = organic label; PEST = pesticide-free label; RGB = regional brand; NAB = national brand; SD = standard deviation.

**Table 5:** Main effect with interaction in trust.

Attributes	Coefficient	SD
PRICE	−0.0323 ***	0.005
ORG	−0.268 ***	0.360
PEST	−0.0697 **	0.365
RGB	0.0279	0.504
NAB	0.151	0.494
ASC	−4.425 ***	0.564
OTRU × ORG	0.238 **	0.101
OTRU × PEST	0.224	0.102
OTRU × RGB	0.267 *	0.141
OTRU × NAB	0.114	0.138
PTRU × ORG	0.159 *	0.084
PTRU × PEST	0.171 **	0.087
PTRU × RGB	−0.224 *	0.119
PTRU × NAB	−0.0395	0.115
χ^2^	1220.76	
P	0.0000	
Log-likelihood	−1614.4741	

Notes: *, **, and *** indicate significance at the 10%, 5%, and 1% levels, respectively. OTRU = Organic trust; PTRU = Pesticide-free trust; ASC = opt-out option; ORG = organic label; PEST = pesticide-free label; RGB = regional brand; NAB = national brand; SD = standard deviation.

**Table 6:** Heterogeneity analysis considering socio-demographics and consumption habits.

Attributes	Coefficient	SD
PRICE	−0.0338 ***	0.00504
ORG	−1.604 **	0.627
PEST	−0.451	0.615
RGB	−0.383	0.836
NAB	−0.0907	0.807
ASC	−10.33 ***	1.477
sex × ORG	0.291 **	0.118
sex × PEST	0.0294	0.116
sex × RGB	0.242	0.155
sex × NAB	0.397 ***	0.153
sex × ASC	1.131 ***	0.264
age × ORG	0.0765	0.0897
age × PEST	−0.0886	0.0867
age × RGB	−0.0474	0.117
age × NAB	−0.0833	0.116
age × ASC	0.420 **	0.17
education × ORG	0.22	0.145
education × PEST	0.504 ***	0.144
education × RGB	−0.238	0.194
education × NAB	−0.123	0.184
education × ASC	0.0309	0.293
marriage × ORG	0.198	0.18
marriage × PEST	−0.185	0.175
marriage × RGB	−0.146	0.238
marriage × NAB	0.0585	0.234
marriage × ASC	0.175	0.372
household size × ORG	0.132 *	0.0693
household size × PEST	0.0599	0.0675
household size × RGB	−0.0441	0.0916
household size × NAB	0.0037	0.0891
household size × ASC	0.362 **	0.146
children × ORG	−0.0123	0.162
children × PEST	0.0458	0.158
children × RGB	0.430 **	0.216
children × NAB	0.105	0.209
children × ASC	0.678 **	0.335
elder × ORG	0.366 ***	0.119
elder × PEST	−0.193 *	0.116
elder × RGB	0.108	0.157
elder × NAB	−0.0531	0.155
elder × ASC	0.262	0.254
income × ORG	0.100 ***	0.0299
income × PEST	0.0187	0.0292
income × RGB	0.104 ***	0.0395
income × NAB	0.0447	0.0386
income × ASC	0.0799	0.0612
frequency × ORG	−0.022	0.0628
frequency × PEST	−0.0343	0.0616
frequency × RGB	0.029	0.0827
frequency × NAB	0.0502	0.0815
frequency × ASC	0.0222	0.134
χ^2^	1206.68	
P	0.000	
Log-likelihood	−1569.3583	

Note: *, **, and *** indicate significance at the 10%, 5%, and 1% levels, respectively. ASC = opt-out option; ORG = organic label; PEST = pesticide-free label; RGB = regional brand; NAB = national brand; SD = standard deviation.

**Table 7:** Estimated WTP: mean coefficients in 0.01 RMB.

Attributes	WTP	SD
PRICE	−6.812 ***	0.645
ORG	1.489 ***	0.171
PEST	0.871 ***	0.260
RGB	0.248	0.187
NAB	0.406 *	0.220
ASC	−4.477 ***	0.160

Notes: * and *** indicate significance at the 10% and 1% levels, respectively. WTP = Willingness to pay; ASC = opt-out option; ORG = organic label; PEST = pesticide-free label; RGB = regional brand; NAB = national brand; SD = standard deviation.

## Data Availability

The data presented in this study are available on request from the corresponding author.
